# Endovascular Embolization for Epistaxis: A Single Center Experience and Meta-Analysis

**DOI:** 10.3390/jcm12226958

**Published:** 2023-11-07

**Authors:** Kareem El Naamani, Charles Morse, Marc Ghanem, Julie Barbera, Abdelaziz Amllay, Grace Severance, Ramon Ruiz, Ahmad Sweid, Michael R. Gooch, Nabeel A. Herial, Pascal Jabbour, Robert H. Rosenwasser, Gurston G. Nyquist, Stavropoula Tjoumakaris

**Affiliations:** 1 Department of Neurological Surgery, Thomas Jefferson University Hospital, Philadelphia, PA 19107, USA; kareem.elnaamani@jefferson.edu (K.E.N.); cem030@jefferson.edu (C.M.); julie.barbera@students.jefferson.edu (J.B.); grace.severance@jefferson.edu (G.S.); ramon.ruiz@students.jefferson.edu (R.R.); sweid.ahmad1@gmail.com (A.S.); michael.gooch@jefferson.edu (M.R.G.); nabeel.herial@jefferson.edu (N.A.H.); pascal.jabbour@jefferson.edu (P.J.); robert.rosenwasser@jefferson.edu (R.H.R.); 2 School of Medicine, Lebansese American University, Beirut 1102-2801, Lebanon; marc.ghanem01@lau.edu; 3 School of Medicine, Hassan II University, Casablanca 8118, Morocco; abdelaziz.amllay@gmail.com; 4 Department of Otolaryngology-Head and Neck Surgery, Thomas Jefferson University Hospital, Philadelphia, PA 19107, USA; gurston.nyquist@jefferson.edu

**Keywords:** epistaxis, embolization, meta-analysis, rebleeding, successful embolization

## Abstract

The optimal treatment for intractable epistaxis is still controversial. Various studies have demonstrated high success rates and low complication rates for endovascular embolization. Herein, the authors report an institutional experience and meta-analysis in terms of efficacy and safety of endovascular embolization of intractable epistaxis. This was a retrospective observational study of 35 patients with epistaxis who underwent 40 embolization procedures between 2010 and 2023. The primary outcome was immediate success defined by immediate cessation of epistaxis at the end of the procedure. Immediate success was achieved in most of the procedures (39, 97.5%). During follow-up, three (7.5%) patients experienced a rebleed. Forty-one studies from 3595 articles were identified for inclusion in the meta-analysis and comprised 1632 patients. The mean pooled age was 57.5 years (95% CI: 57.2–57.8) and most patients were males (mean: 70.4, 95% CI: 69.8–71.0). Immediate success was achieved at a pooled mean of 90.9% (95% CI: 90.4–91.4) and rebleeding was observed at a pooled mean of 17% (95% CI: 16.5–17.5). In conclusion, endovascular embolization proved to be both safe and effective in treating intractable epistaxis carrying a low risk of post-operative stroke.

## 1. Introduction

Epistaxis is defined as bleeding from the nasal fossae and occurs in around 60% of the population. Fortunately, only 6% of cases require medical attention [1,2]. Despite being mostly self-limited, a proportion may be an emergency due to the blood volume, recurrent episodes, or patients’ comorbidities. Approximately 90% of cases arise from the anterior nasal septum, termed as anterior epistaxis, which is readily controlled with conservative methods. On the other hand, posterior epistaxis is less common and more likely requires aggressive management [3]. Management options range in invasiveness from anterior rhinoscopy with silver nitrate cautery with/without packing to endoscopic guided electrocoagulation with/without posterior nasal packing.

Various treatment strategies are described for intractable epistaxis. Posterior nasal packing is associated with higher hospitalization cost and risk of complications such as septal hematomas, abscesses, septicemia, pressure necrosis, and posterior displacement of the pack [4,5]. The complications rate for nasal packing ranges from 2% to 68%, with a 25–52% failure rate. Thus, nasal packing is a less desirable option for treating intractable epistaxis [6,7,8,9].

Historically, surgical ligation of the internal maxillary artery has been the treatment of choice for intractable epistaxis [10,11,12]. Subsequently, with advances in technology and endoscopic procedures, the endoscopic sphenopalatine artery (SPA) ligation has gained favor [2]. Moreover, advancement in modern technologies such as microcatheters allowed for a safe and precise targeted embolization. Favorable outcomes resulted in endovascular embolization being increasingly recommended as the primary treatment of choice [3,6,9,12,13,14,15]. The nasal vasculature arises from the external carotid artery (ECA) and internal carotid artery (ICA) branches. The ECA contributes most of the blood supply via distal internal maxillary artery branches (IMA), namely sphenopalatine arteries (SPA) and greater palatine arteries, with additional supply from facial arteries. The ICA contributes to the anterior and posterior ethmoidal arteries through the distal ophthalmic artery.

The optimal treatment for intractable epistaxis is still controversial. Various studies have demonstrated high success rates and low complication rates for endovascular embolization [3,6,12]. One dreaded complication of embolization is stroke, which may arise due to extracranial–intracranial collaterals, which are often difficult to assess angiographically. Thus, the aim of our study was to provide an institutional experience with endovascular embolization of epistaxis and summarize the literature pertaining to outcomes and safety profile.

## 2. Materials and Methods

### 2.1. Case Series

#### 2.1.1. Patient Population

This was a retrospective observational study of patients with posterior epistaxis who underwent embolization procedures between 2010 and 2023. Patients were identified by searching a database maintained at the neuro-interventional department. Medical charts were reviewed for baseline characteristics (gender, smoking status, hypertension, bleeding diathesis, blood thinner use, and recreational drug use), previous surgical treatment, details of embolization treatment (number of vessels embolized, laterality, arteries embolized, intraprocedural complications, microcatheter used, and embolisate material), postoperative complications (ischemic stroke, vision change, retroperitoneal hemorrhage, groin hematoma, failed arterial access, palatal ulcers, nasal/facial pain, nasal/facial/palatal numbness), outcomes (immediate success, short term failure, and long term failure), and mortality and follow-up duration. Bleeding diathesis included any diseases that causes blood thinning. Recreational drugs are drugs consumed for pleasure (e.g., cocaine, heroin, etc.) The study protocol was reviewed and approved by the Institutional Review Board as part of the endovascular database on 5 July 2020 (ID: #12D. 534). Following our institutional guidelines, all protected health information was removed, and individual patient consents were not required for the analysis of this case series.

#### 2.1.2. Procedure

Arterial access is usually achieved using a radial or a femoral artery approach. A 6-Fr Benchmark and Berenstein catheters (Boston Scientific, Marlborough, MA, USA) were used with the aid of a 0.038 Terumo guidewire. The external carotid artery was catheterized bilaterally and was carefully evaluated, explicitly looking for dangerous anastomoses with the ICA or orbit, and to assess for the presence of a hyper-vascular mucosal blush, robust arterial filling, pseudoaneurysm, or arteriovenous malformation. Target vessels were supra-selectively catheterized with Echelon-1 (Medtronic, Dublin, Ireland) or SL10 (Stryker Neurovascular, Kalamazoo, MI, USA) microcatheters over a Synchro-10 micro-guidewire (Stryker Neurovascular, Kalamazoo, MI, USA). Embolization was carried out with either Onyx (Onyx Liquid Embolic System, Micro Therapeutics, Inc., Irvine, CA, USA), or particles (microparticles, density changes between procedures), or both. Care was taken to avoid reflux of the embolisate material into the more proximal arterial territories. A post-embolization control angiogram was routinely obtained to assess the effectiveness of the occlusion. The packs were removed in the NICU under direct supervision by the Otorhinolaryngological service within 24 h from the completion of embolization.

#### 2.1.3. Outcomes

Endovascular embolization was performed as a rescue procedure following the failure of endoscopic arterial ligation in the vast majority of patients. Briefly, outcomes were stratified into immediate success defined as adequate hemostasis at the completion of the procedure and encompassing up to 24 h. Rebleeding included patients that did not achieve success from the first try, or those in which immediate success was achieved but rebled later. Ischemic stroke was also stratified into minor and major based on a change of ≥4 points in the National Institute of Health Stroke Scale. Facial pain was considered a sequela of the embolization procedure if it persisted at the follow-up clinical visit.

### 2.2. Meta Analysis

#### 2.2.1. Literature Search

Using PubMed, Web of Science, and Cochrane Central Register of Controlled Trials, a systemic literature review was performed in accordance with the Preferred Reporting Items for Systemic Reviews and Meta-Analyses (PRISMA) statement on 15 August 2022. In all three search engines, we started with the search words “epistaxis” OR “nosebleed” OR “nasal hemorrhage” OR “nasal bleed” OR “intractable epistaxis” AND “embolization” OR “endovascular embolization”. No protocol was utilized in this review. Initially, articles were filtered based on the title and abstract. After that, one author reviewed the full texts to determine inclusion eligibility and to review the references for additional studies. Duplicates from the different search engines were removed. The inclusion criteria were studies reporting outcomes of endovascular embolization of epistaxis. Studies in languages other than English, studies providing outcomes of both embolization and surgery combined, and studies not specifying outcomes were excluded. A “-” was assigned to a data point that we could not extrapolate any information on from the studies.

#### 2.2.2. Outcomes

The primary outcome was immediate success defined by immediate cessation of epistaxis at the end of the procedure. Secondary outcomes included rebleeding rate and complication rate. Rebleeding rate included patients who failed initial embolization and patients who achieved immediate success but rebled afterwards. Complications were divided into major complications including stroke, necrosis, facial nerve palsy, and ophthalmic injury, and minor complications including headache, mental status alteration, facial edema, and facial numbness.

#### 2.2.3. Statistical Analysis

Statistical analyses were performed using the Stata software (version 17; StataCorp; College Station, TX, USA). The meta-analysis was conducted using the ‘metaprop’ package within R, allowing for the estimation of a pooled proportion while considering study-specific weights based on sample sizes. The choice of the meta-analysis model, either random effects or fixed effects, depended on the assessment of heterogeneity between studies. Heterogeneity was evaluated both using the I-squared statistic (a higher value indicates a greater degree of variability among study results), and Tau-squared (τ^2^) (provides an estimate of the between-study variance). The pooled means were computed by assigning appropriate weights to individual data points, accounting for factors such as sample sizes. This approach ensures that the summary estimate effectively estimates means from multiple sources while giving more prominence to studies with larger sample sizes.

## 3. Results

### 3.1. Case Series

The total study cohort was composed of 35 patients who underwent 40 procedures. A total of 30 patients (85.7%) underwent one procedure, 4 patients (11.4%) underwent 2 procedures, and 1 patient (2.8%) underwent 3 procedures (1 of which was done at an outside hospital) (Table 1).

The mean age of the cohort was 66.3 years ± 15.4, 95% CI: 61.9–72.5, and the majority were males (*n* = 19, 54.3%). Regarding past medical and social history, 2 patients were smokers (5.7%), 20 patients presented with hypertension (57.1%), and 4 patients used recreational drugs (11.4%). Moreover, 14 patients were on antiplatelets (40%), divided into 10 patients on a single antiplatelet medication (28.6%), and 4 patients on dual antiplatelet therapy (11.4%). A total of 11 patients were also on anticoagulation (31.4%) and 4 patients suffered from bleeding diathesis (11.4%). A total of 33 patients (94.3%) underwent previous surgical treatments for epistaxis.

The mean time between a failed SPA procedure and embolization was 19.1 days ± 31.8, 95% CI: 31.8–6.42 with 5 patients undergoing embolization 5 years, 4 years, 1 year, and 8 months after their failed SPA procedures.

The mean number of vessels embolized was 1.9 ± 0.9, 955 CI: 1.6–2.2. Laterality of embolization procedures were: 9 vessels on the left side (22.5%), 7 on the right side (17.5%), and 24 bilateral (60%). Out of the total 44 vessels, the internal maxillary artery was the most common embolized vessel (38, 86.4%), followed by the facial artery (6, 13.6%). The most common distal catheter used was SL-10 (20, 50%). As for the embolisate material, Onyx was used in 18 procedures (45%), particles were used in 8 procedures (20%), and a combination of both was used in 14 procedures (35%).

There were no intraprocedural complications; however, 20 patients developed post-procedural complications. These included eight cases of transient nasal/facial pain (20%), five cases of transient vision changes (12.5%) varying from diplopia to vision loss, five cases suffering from nasal/facial/palatal numbness that resolved (12.5%), one case of groin hematoma (2.5%), and one case of palatal ulceration that resolved (2.5%). None of the patients developed postprocedural ischemic stroke or retroperitoneal hemorrhage (0%). Immediate success was achieved in most of the procedures (39, 97.5%). In the first 24 h post-op, three (7.5%) patients rebled.

### 3.2. Meta-Analysis

#### 3.2.1. Patient Demographics

**Figure 1:**
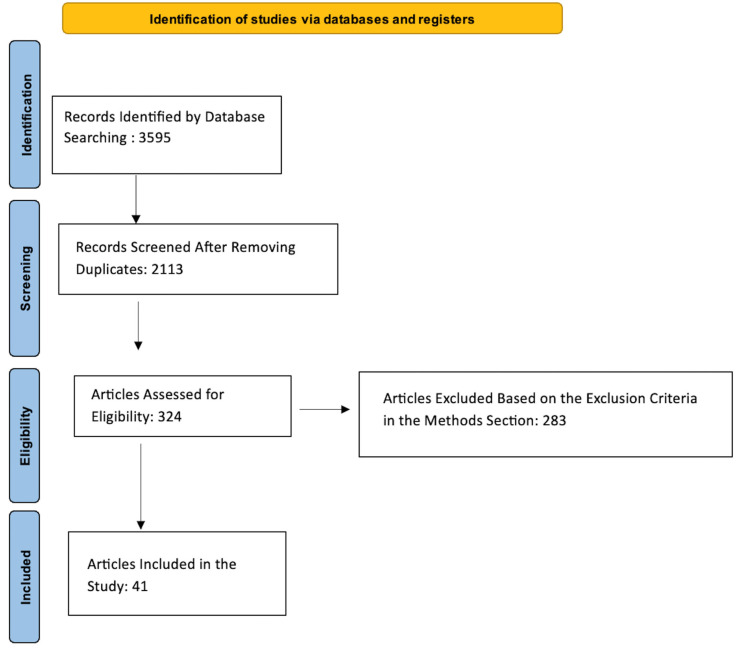
Flow diagram showing the systemic review process.

Out of 3595 articles, a total of 41 articles were included in the study and comprised 1632 patients (Table 2, Figure 1).

The mean pooled age was 57.5 years (95% CI: 57.2–57.8) and most patients were males (mean: 70.4, 95% CI: 69.8–71).

#### 3.2.2. Outcomes

**Figure 2:**
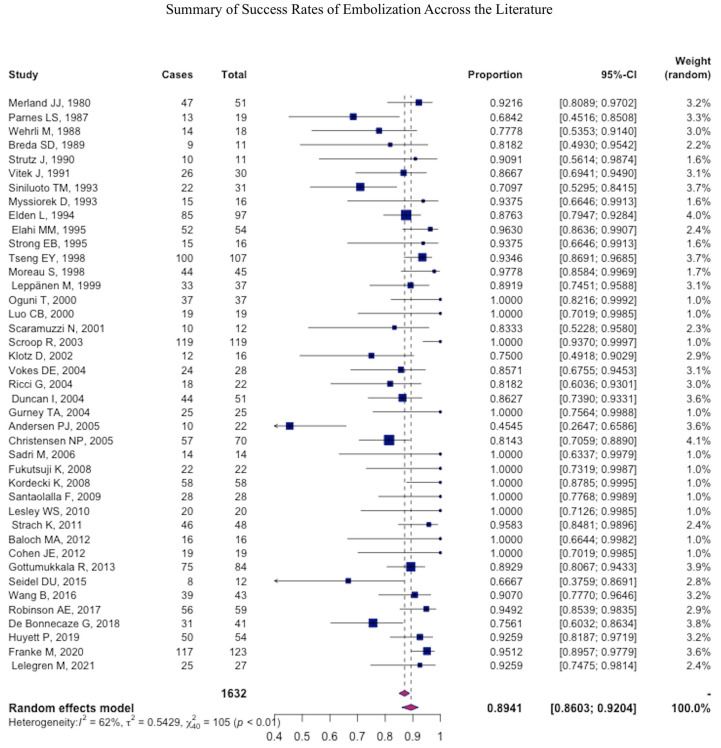
Forest plot summarizing the success rate across the literature.

**Figure 3:**
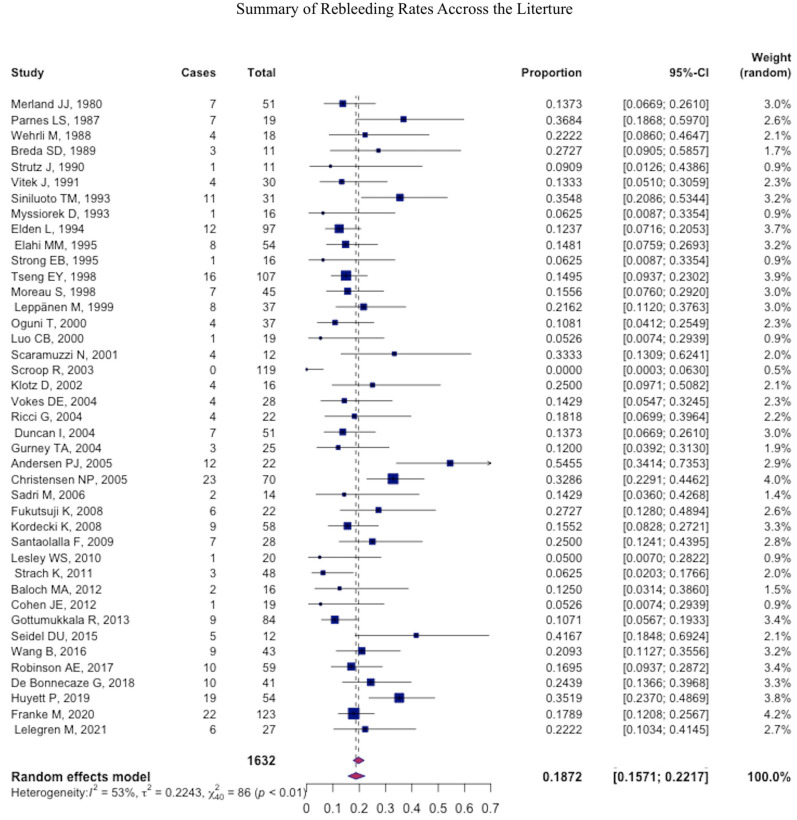
Forest plot summarizing the rebleeding rate across the literature.

After the procedure, immediate success was achieved at a pooled mean of 90.9% (95% CI: 90.4–91.4) and rebleeding was observed at a pooled mean of 17% (95% CI: 16.5–17.5) (Table 3) (Figure 2 and Figure 3).

#### 3.2.3. Complications

**Figure 4:**
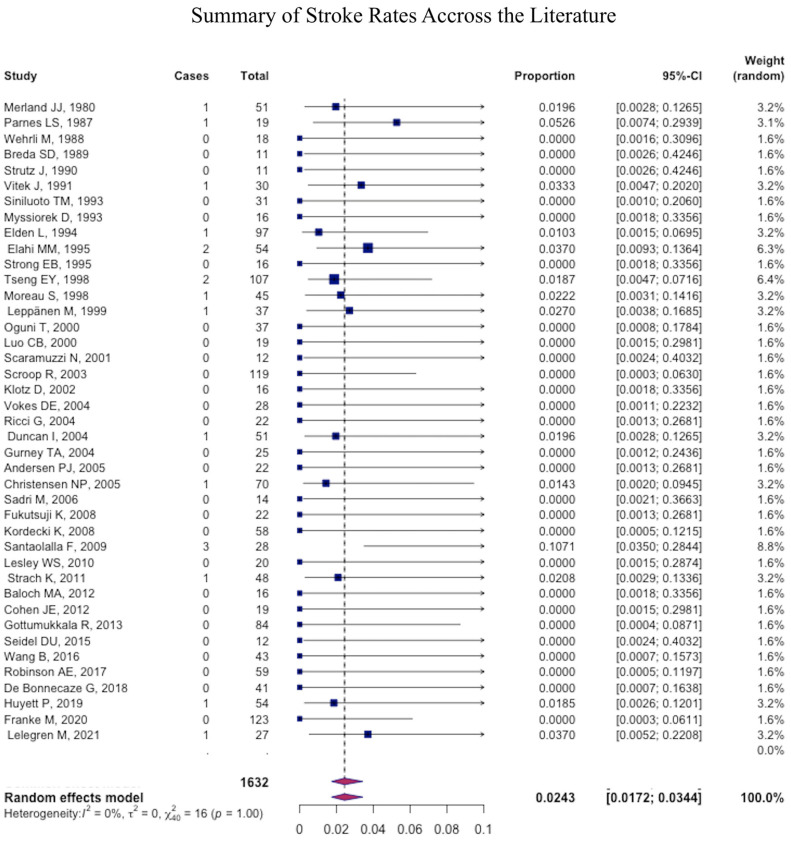
Forest plot summarizing the rate of stroke across the literature.

**Figure 5:**
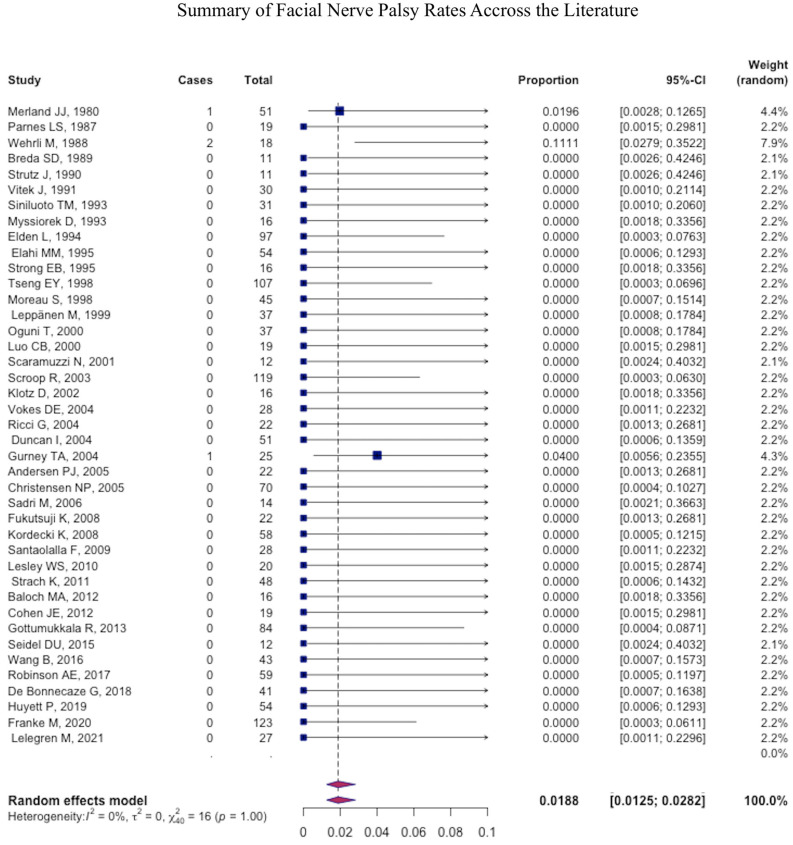
Forest plot summarizing the rate of facial nerve palsy across the literature.

**Figure 6:**
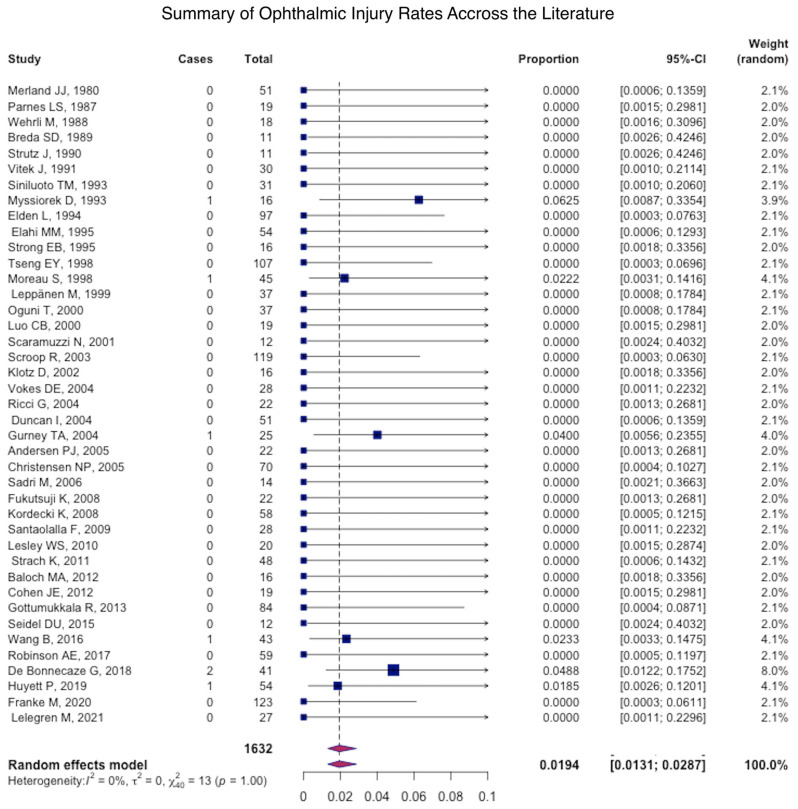
Forest plot summarizing the rate ophthalmic injury across the literature.

The most common major complication was necrosis (pooled mean: 1.2% (95% CI: 1.1–1.4), followed by stroke (pooled mean: 1.1% (95% CI: 1.0–1.2) (Figure 4), ophthalmic injury (pooled mean: 0.4% (95% CI: 0.4–0.5), and facial nerve palsy (pooled mean: 0.2% (95% CI: −0.2–0.3) (Figure 5 and Figure 6) (Table 4).

As for minor complications, the most common was facial pain (pooled mean: 13.1% (95% CI: 12–14.2), followed by headache (pooled mean: 2.8% (95% CI: 2.5–3.2), facial numbness (pooled mean: 1.4% (95% CI: 1.2–1.6), facial edema (pooled mean: 0.4% (95% CI: −0.4–0.5), and mental status change (pooled mean: 0.4% (95% CI: −0.3–0.5) (Table 5).

## 4. Discussion

Epistaxis is a health problem that occurs in approximately 60% of the population, 6% of which require medical attention [1,2]. Among those who require medical attention, the majority resolved with nasal packing [12]. However, when conservative measures fail to control the hemorrhage, endovascular embolization or surgical ligation of the offending vessels is warranted. Surgical ligation of the IMA has been performed since the middle of the 1960s as the treatment of choice for intractable epistaxis; however, since 1974, endovascular embolization has emerged as a viable, well-tolerated, and effective treatment alternative or adjunct treatment for intractable epistaxis [1,3,6,7,8,9,10,11,12,13,14,15,18,20,23,24,29,30,31,34,35,37,38,42,45,48,49,50,51,52].

The immediate success rate in our case series was 97.5%. Three patients developed a rebleed, of which two required re-embolization in the first 24 h. The rate of rebleed reported in our series (7.5%) was lower than the pooled mean of rebleeding (18.8%) and lies in the range reported by the literature (0–55%) [3,17,19,21,32,36,38,47]. Moreover, our immediate and success rates were comparable to the literature (45–100%) [6,7,16,17,28,39,42,44]. These rates also compare favorably with the reported average success rate of 87% for surgical ligation [2,14]. It is paramount to note that despite favorable outcomes, this a challenging cohort of patients, of whom 33/40 had previously failed SPA ligation. The main reasons for SPA ligation failure are slipping of the surgical clips and failure to identify all branches of the SPA [53]. Revision surgery after failure of SPA ligation poses high morbidity and mortality rates [53]. Without any doubt, these results demonstrate the efficacy of endovascular embolization in controlling intractable epistaxis. Potential explanations for re-bleeding following embolization include failure to embolize the targeted vessel(s) or a new bleeding source [10,14]. In our series, this failure may also stem from having embolization as a rescue treatment following the failure of endoscopic artery ligation. This suggests that patients referred for embolization in our study represent a high-risk group with increased risk for failure. An example is patients that require chronic anticoagulation or anti-platelet therapy, represented in at least 70% of our patient population. This may introduce a treatment bias that is predisposed to failure and complications. Moreover, most of our patients were hypertensive (57%) or on blood thinners (71%). These comorbidities increase the risk of failure or complications. Several studies have confirmed the correlation between blood thinners and increased risk of failure after endovascular embolization in treating intractable epistaxis [3,42,45].

Although the endovascular therapy of epistaxis is effective, a potential risk for complications remains associated with this technique. The major complications reported in the literature include stroke, permanent blindness, facial nerve palsy, and necrosis of soft tissues, while the minor complications are largely transient and include facial pain, headaches, paresthesia/numbness, groin pain, and groin hematoma [6,11,24,49].

The incidence of minor complications in our case series was 40%, mostly of transient nasal/facial pain (20%). All complications encountered in our study resolved within one month of the procedure. The minor complication rate reported in our study is within the 6–45% range reported in other large case series [11,23,24,35]. Certain studies have considered re-bleeding as a complication, while others have included additional complications such as pain and numbness, as was done in the current study. Gottumukkala et al. demonstrated that as the number of embolized ECA branches increases from one to four, the risk of recurrent bleeding is significantly reduced from 25% to 0% [42]. In contrast, the rate of minor complications increases from 0 to 56%. In our study, the mean number of embolized vessels was 1.9. Unfortunately, published data regarding the optimal technique for epistaxis embolization are scarce.

None of our patients have experienced any major embolic complications (0%), which compares favorably with what is reported in the literature. In most large series, the rate of ischemic stroke ranged from 0–2% [7,12,24]. Reported rates of TIA and blindness were similarly low. Brinjikji et al. reported a stroke incidence at less than 1% after retrospectively reviewing 64,289 patients [12]. Embolization-related major complications are thought to be due to an over-embolization, the use of particles that are too small, or by reflux into ICA. The hypothesis suggests that forceful injection of embolisate increases the pressure during embolization, causing pre-existing anastomoses to open, which results in the accidental embolization of the ICA or the ophthalmic artery [6,8].

### Limitations

The limitations include the single-center retrospective design, which can assess the associations and not causality, the small number of patients, and the selection bias in the referral pattern for endovascular embolization. Additionally, all studies in our meta-analysis were retrospective and carry significant risk of bias. The definition of outcomes differed between the studies, and follow-up duration for rebleeding was variable. Because of this, underestimation of minor complications was a strong possibility.

## 5. Conclusions

In conclusion, endovascular embolization has been shown to be both safe and effective in treating intractable epistaxis and carries a low risk of post-operative ischemic strokes. Conservative management should be exhausted before opting for endovascular embolization because this modality of treatment, though rarely, carries a rare risk of morbidity.

## Figures and Tables

**Table 1:** Baseline patients and procedural characteristics.

Baseline Characteristics	N (%) Mean (SD, 95% CI, Range)
Number of patients	35
Number of procedures	40
Number of Arteries embolized	44
Number of procedures per patient	
30 patients	1 procedure
4 patients	2 procedures
1 patient	3 procedures
Age	66.3 ± 15.4; 95% 61.96–72.56
Gender (Male)	19 (54.3)
Smokers	2 (5.7)
Hypertension	20 (57.1)
Antiplatelet	14 (40)
Single	10 (28.6)
Dual	4 (11.4)
Anticoagulation	11 (31.4)
Bleeding Diathesis	4 (11.4)
Recreational Drugs	4 (11.4)
Previous Surgical Treatment	33 (94.3)
Number of vessels embolized	1.9 ± 0.9; 95% CI 1.6–2.2
Time From failed SPA Ligation to embolization (days)	19.1 ± 31.8, 95% CI: 31.8–6.42, 1–148
Laterality	
Left	9 (22.5)
Right	7 (17.5)
Bilateral	24 (60)
Arteries Embolized	
Internal Maxillary Artery	38 (86.4)
Facial Artery	6 (13.6)
Procedural Complications	0
Catheters Used	
Scepter	3 (7.5)
SL-10	20 (50)
Echelon	6 (15)
Duo Microcatheter	5 (12.5)
Marathon	4 (10)
Prowler	1 (2.5)
Turbo Track 18	1 (2.5)
Embolisate	
Onyx	18 (45)
Particles	8 (20)
Combination	14 (35)
Complications	
Stoke (minor, major)	0
Vision Change	5 (12.5)
Retroperitoneal Hemorrhage	0
Groin Hematoma	1 (2.5)
Failed Arterial Access	0
Palatal Ulcers	1 (2.5)
Nasal/Facial Pain	8 (20)
Nasal/Facial/Palatal Numbness	5 (12.5)
Immediate Success	39 (97.5)
Rebleed	3 (7.5)
Mortality	0
Length of Follow-up (months)	17.85 ± 21.79; 95% CI 10.4–25.3

**Table 2:** Demographic and baseline characteristics of studies comprising patients endovascularly treated for epistaxis. ^†^ Pooled means and proportions with corresponding 95% confidence intervals.

Study/Year	Patients, N	Age, Mean	Male (%)
Merland et al., 1980 [16]	51	-	-
Parnes et al., 1987 [17]	19	-	-
Wehrli et al., 1988 [18]	18	57	61.1
Breda et al., 1989 [19]	11	56	54.5
Strutz et al., 1990 [20]	11	48	45.5
Vitek et al., 1991 [9]	30	62	70
Siniluoto et al., 1993 [21]	31	49	83.9
Myssoirek et al., 1993 [22]	16	40	68.8
Elden et al., 1994 [7]	97	53	66
Elahi et al., 1995 [23]	54	53	63
Strong et al., 1995 [14]	16	61	81.3
Tseng et al., 1998 [24]	107	55	-
Moreau et al., 1998 [25]	45	49	75.6
Leppänen et al., 1999 [11]	37	53	78.4
Oguni et al., 2000 [26]	37	57	83.8
Luo et al., 2000 [27]	19	38	78.9
Scaramuzzi et al., 2001 [28]	12	51	83.3
Klotz et al., 2002 [29]	16	-	-
Scroop et al., 2003 [30]	119	-	83.2
Vokes et al., 2004 [10]	28	55	64.3
Ricci et al., 2004 [31]	22	62	63.6
Duncan et al., 2004 [32]	51	54	54.9
Gurney et al., 2004 [33]	25	67	60
Anderson et al., 2005 [34]	22	59	72.7
Christensen et al., 2005 [35]	70	59	58.6
Sadri et al., 2006 [36]	14	57	85.7
Fukutsuji et al., 2008 [6]	22	57	95.5
Kordecki et al., 2008 [37]	58	-	
Santaolalla et al., 2009 [38]	28	60	89.3
Lesley et al., 2010 [39]	20	63	65
Strach et al., 2011 [8]	48	57	75
Baloch et al., 2012 [40]	16	51	87.5
Cohen et al., 2012 [41]	19	61	89.5
Gottumukkala et al., 2013 [42]	84	64	56
Seidel et al., 2015 [15]	12	58	75
Wang et al., 2016 [43]	43	46	88.4
Robinson et al., 2017 [3]	59	59	72.9
de Bonnecaze et al., 2018 [44]	41	66	-
Huyett et al., 2019 [45]	54	65	66.7
Franke et al., 2020 [46]	123	66	65
Lelegren et al., 2021 [47]	27	64	59.3
Pooled Estimate ^†^, CI	-	57.5 (57.2–57.8)	70.4 (69.8–71.0)

**Table 3:** Rate of immediate success and rebleeding.

Study/Year	Immediate Success (%)	Rebleed (%)
Merland et al., 1980 [16]	92.2	13.7
Parnes et al., 1987 [17]	68.4	36.8
Wehrli et al., 1988 [18]	77.8	22.2
Breda et al., 1989 [19]	81.8	27.3
Strutz et al., 1990 [20]	90.9	9.1
Vitek et al., 1991 [9]	86.7	13.3
Siniluoto et al., 1993 [21]	71	35.4
Myssoirek et al., 1993 [22]	93.8	6.3
Elden et al., 1994 [7]	87.6	12.4
Elahi et al., 1995 [23]	96.3	14.8
Strong et al., 1995 [14]	93.8	6.3
Tseng et al., 1998 [24]	92.5	15
Moreau et al., 1998 [25]	97.8	15.6
Leppänen et al., 1999 [11]	89.2	21.6
Oguni et al., 2000 [26]	100	10.8
Luo et al., 2000 [27]	100	5.3
Scaramuzzi et al., 2001 [28]	83.3	33.3
Klotz et al., 2002 [29]	75	25
Scroop et al., 2003 [30]	100	0
Vokes et al., 2004 [10]	85.7	14.3
Ricci et al., 2004 [31]	81.8	18.2
Duncan et al., 2004 [32]	86.3	13.7
Gurney et al., 2004 [33]	100	12
Anderson et al., 2005 [34]	45.5	54.5
Christensen et al., 2005 [35]	81.4	32.9
Sadri et al., 2006 [36]	100	14.3
Fukutsuji et al., 2008 [6]	100	27.3
Kordecki et al., 2008 [37]	100	15.5
Santaolalla et al., 2009 [38]	100	25
Lesley et al., 2010 [39]	100	5
Strach et al., 2011 [8]	95.8	6.3
Baloch et al., 2012 [40]	100	12.5
Cohen et al., 2012 [41]	100	5.3
Gottumukkala et al., 2013 [42]	89.3	10.7
Seidel et al., 2015 [15]	66.7	41.7
Wang et al., 2016 [43]	90.7	20.9
Robinson et al., 2017 [3]	94.9	16.9
de Bonnecaze et al., 2018 [44]	75.6	24.4
Huyett et al., 2019 [45]	92.6	35.2
Franke et al., 2020 [46]	95.1	17.9
Lelegren et al., 2021 [47]	92.6	22.2
Pooled Estimate (CI)	90.9 (90.4–91.4)	17 (16.5–17.5)

**Table 4:** Summary of major complications.

Study/Year	Stroke (%)	Ophthalmic Injury (%)	Facial Nerve Palsy (%)	Necrosis (%)
Merland et al., 1980 [16]	2	0	2	0
Parnes et al., 1987 [17]	5.3	0	0	0
Wehrli et al., 1988 [18]	0	0	11.1	11.1
Breda et al., 1989 [19]	0	0	0	9.1
Strutz et al., 1990 [20]	0	0	0	0
Vitek et al., 1991 [9]	3.3	0	0	0
Siniluoto et al., 1993 [21]	0	0	0	0
Myssoirek et al., 1993 [22]	0	6.3	0	0
Elden et al., 1994 [7]	1	0	0	2.1
Elahi et al., 1995 [23]	3.7	0	0	0
Strong et al., 1995 [14]	0	0	0	0
Tseng et al., 1998 [24]	1.9	0	0	0
Moreau et al., 1998 [25]	2.2	2.2	0	0
Leppänen et al., 1999 [11]	2.7	0	0	0
Oguni et al., 2000 [26]	0	0	0	0
Luo et al., 2000 [27]	0	0	0	0
Scaramuzzi et al., 2001 [28]	0	0	0	0
Klotz et al., 2002 [29]	0	0	0	6.3
Scroop et al., 2003 [30]	0	0	0	0
Vokes et al., 2004 [10]	0	0	0	0
Ricci et al., 2004 [31]	0	0	0	0
Duncan et al., 2004 [32]	2	0	0	0
Gurney et al., 2004 [33]	0	4	4	0
Anderson et al., 2005 [34]	0	0	0	4.5
Christensen et al., 2005 [35]	1.4	0	0	0
Sadri et al., 2006 [36]	0	0	0	14.3
Fukutsuji et al., 2008 [6]	0	0	0	0
Kordecki et al., 2008 [37]	0	0	0	0
Santaolalla et al., 2009 [38]	10.7	0	0	0
Lesley et al., 2010 [39]	0	0	0	0
Strach et al., 2011 [8]	2.1	0	0	2.1
Baloch et al., 2012 [40]	0	0	0	0
Cohen et al., 2012 [41]	0	0	0	0
Gottumukkala et al., 2013 [42]	0	0	0	1.2
Seidel et al., 2015 [15]	0	0	0	0
Wang et al., 2016 [43]	0	0	0	2.3
Robinson et al., 2017 [3]	0	0	0	0
de Bonnecaze et al., 2018 [44]	0	0	0	12.2
Huyett et al., 2019 [45]	1.9	0	0	7.4
Franke et al., 2020 [46]	0	0	0	0
Lelegren et al., 2021 [47]	3.7	0	0	0
Pooled Estimate	1.1 (1.0–1.2)	0.4 (0.4–0.5)	0.2 (0.2–0.3)	1.2 (1.1–1.4)

**Table 5:** Summary of minor complications.

Study/Year	Facial Pain (%)	Headache (%)	Mental Status Change (%)	Facial Numbness (%)	Facial Edema (%)
Merland et al., 1980 [16]	-	-	-	-	-
Parnes et al., 1987 [17]	31.6	0	0	0	0
Wehrli et al., 1988 [18]	33.3	0	0	16.7	0
Breda et al., 1989 [19]	0	0	0	0	0
Strutz et al., 1990 [20]	0	0	0	0	0
Vitek et al., 1991 [9]	0	0	0	0	0
Siniluoto et al., 1993 [21]	96.8	0	0	0	0
Myssoirek et al., 1993 [22]	68.8	0	0	6.3	0
Elden et al., 1994 [7]	20.6	0	0	0	0
Elahi et al., 1995 [23]	3.7	0	0	1.9	0
Strong et al., 1995 [14]	0	0	0	0	0
Tseng et al., 1998 [24]	5.6	2.8	4.7	0.9	0.9
Moreau et al., 1998 [25]	2.2	0	0	0	0
Leppänen et al., 1999 [11]	0	0	0	8.1	0
Oguni et al., 2000 [26]	24.3	18.9	0	0	0
Luo et al., 2000 [27]	10.5	0	0	0	0
Scaramuzzi et al., 2001 [28]	16.7	0	0	0	0
Klotz et al., 2002 [29]	-	-	-	-	-
Scroop et al., 2003 [30]	0	0	0	0	0
Vokes et al., 2004 [10]	3.6	3.6	0	3.6	0
Ricci et al., 2004 [31]	0	0	0	0	0
Duncan et al., 2004 [32]	3.9	5.9	0	0	0
Gurney et al., 2004 [33]	0	0	0	4	0
Anderson et al., 2005 [34]	68.1	0	0	9.1	0
Christensen et al., 2005 [35]	-	-	-	-	-
Sadri et al., 2006 [36]	0	0	0	0	0
Fukutsuji et al., 2008 [6]	27.3	9.1	0	13.6	4.5
Kordecki et al., 2008 [37]	0	15.5	0	0	5.2
Santaolalla et al., 2009 [38]	17.9	25	0	0	0
Lesley et al., 2010 [39]	5	0	0	0	0
Strach et al., 2011 [8]	-	-	-	-	-
Baloch et al., 2012 [40]	6.3	0	0	0	0
Cohen et al., 2012 [41]	0	0	0	0	0
Gottumukkala et al., 2013 [42]	20.2	3.6	0	0	1.2
Seidel et al., 2015 [15]	0	0	0	0	0
Wang et al., 2016 [43]	30.2	0	0	0	0
Robinson et al., 2017 [3]	-	-	0	0	0
de Bonnecaze et al., 2018 [44]	12.2	0	0	0	0
Huyett et al., 2019 [45]	7.4	0	0	3.7	0
Franke et al., 2020 [46]	-	-	-	-	-
Lelegren et al., 2021 [47]	0	0	0	0	0
Pooled Estimate	13.1 (12–14.2)	2.8 (2.5–3.2)	0.4 (−0.3–0.5)	1.4 (1.2–1.6)	0.4 (−0.4–0.5)

## Data Availability

The relevant anonymized patient-level data are available on reasonable request from the authors.
